# Correction: Receptive field center-surround interactions mediate context-dependent spatial contrast encoding in the retina

**DOI:** 10.7554/eLife.100599

**Published:** 2024-06-26

**Authors:** Maxwell H Turner, Gregory W Schwartz, Fred Rieke

**Keywords:** Rhesus macaque, Other

Turner MH, Schwartz GW, Rieke F. 2018. Receptive field center-surround interactions mediate context-dependent spatial contrast encoding in the retina. *eLife* **7**:e38841. doi: 10.7554/eLife.38841.

Published 6 September 2018

A reader brought to our attention an error in equation 12 described in the materials and methods section.

Equation 12 should read:

$$RI=1-\;\frac{r_{0}-r_{-}}{r_{+}-r_{0}}$$

The original version of equation 12, shown here for reference, read:

$$RI=1-\;\frac{r_{0}-r_{-}}{r_{0}-r_{+}}$$

This error was introduced when preparing the methods section of the manuscript. The correct equation was used in the analysis code that produced the results shown in the paper (see relevant source code here, line 255: https://github.com/mhturner/CenterSurroundInteractions/blob/master/FigureCode/doCSLNAnalysis.m).

The article has been corrected accordingly.

